# Types of Psoriasis and Their Effects on the Immune System

**DOI:** 10.7759/cureus.29536

**Published:** 2022-09-24

**Authors:** Anushka Dhabale, Shailesh Nagpure

**Affiliations:** 1 Pathology and Laboratory Medicine, Jawaharlal Nehru Medical College, Nagpur, Maharashtra, IND; 2 Pharmacology, Jawaharlal Nehru Medical College, Datta Meghe Institute of Medical Sciences, Wardha, IND

**Keywords:** plaque psoriasis, immune system, pustular psoriasis, types of psoriasis, psoriasis

## Abstract

Psoriasis is a chronic inflammatory skin disease which is identified by the appearance of erythematous that is clearly demarcated, scaly plaques. It is a skin disease seen regularly around the elbow, scalp, trunk, and also on the knees. Psoriasis is a commonly occurring chronic disease with no cure. In psoriasis, which is thought to be an immune system-related problem, the cells of the skin grow quicker than normal cells. The rapid turnover of cells is responsible for the dry scaly patches seen clinically, also called plaque type of psoriasis. The etiopathogenesis of psoriasis is not yet fully understood. It is considered an outcome of some alteration of the cells in the immune system, which fights infections, but here, it attacks healthy cells, which is the problem. Researchers believe both environmental and genetic factors play a role. It is commonly known that psoriasis is not contagious as it does not spread by air or water. There is a chance of increasing the risk of acquiring psoriasis, also worsening the disease severity by smoking and tobacco consumption. Anyone irrespective of age or gender can develop psoriasis. Psoriasis is divided into various kinds: plaque, nail type, guttate, or inverse, also pustular. The most commonly occurring type of psoriasis is plaque psoriasis, seen with itchy, dry, scales covering patches of skin that are raised.

## Introduction and background

Psoriasis is an immune-mediated skin disease with a genetic predisposition. There is an involvement of the interaction of adaptive and innate immunity, which is the main pathological mechanism in this disease. Cytokines, which are secreted, mediate the interaction of the T cells with the cells of dendrites, keratinocytes, and macrophages [1]. Biologists over the past decade have developed and approved blockers for interleukin IL-23, tumour necrotic factor α, and IL-17 for psoriasis treatment [2]. This disease is an immune systems-related disease of joints and also of skin, which is recurrent, chronic, and common. It has a considerable huge negative impact on various aspects of an affected patient's health, like emotional, psychosocial, and physical well-being [2].

One of the main determinants of expression of the disease is the carriage of the HLA-Cw6 and environmental triggers such as beta-haemolytic infection caused by *Streptococcus *in the early stage of psoriasis, like if it begins before 40 years of age [3]. The cells that are antigen-presenting are present in the skin and secrete the IL-12 and IL-23, which ultimately activate type 1 (Th1) and type 17 (Th17) T helper cells to produce a cellular type of immune response. The cutaneous findings which are observed in this disease are due to the development of a state of chronic inflammation, altered hyperproliferation of epidermis, apoptosis, differentiated mechanism, and neo-angiogenesis which is caused by different types of cytokines such as tumour necrosis factor (TNF) α [4]. 

There can be dysfunction in the immune systems, particularly in autoimmune diseases, which are caused by specific triggers that vary among individuals. Whereas in psoriasis, it most commonly may include trauma of the skin, like bites of insects, scratches, and sunburn. Stress can also be considered as one of the triggers. An inflammatory response is accidentally generated in the activated immune system in psoriasis. The immune system works against or attacks the healthy cells as if they resemble foreign invading harmful pathogens. Here, signalling molecules are produced in excess, as well as the helper variant of T type of lymphocytes or T kind of cells, which are the white cells of blood, that become irregularly active. The blood vessels present in the skin widen due to the action of cytokine molecules. So thereafter, there will be an accumulation of keratinocytes and white blood cells, which will in turn make the outermost skin layer grow much quicker than the normal one. In the usual scenario, a person without psoriasis would take up to 3-4 weeks for cell maturation, migration to the skin surface, dividing, and also sloughing off, whereas in psoriasis, for the same events, it takes just 3-7 days. The outcome of this is that there is a thick skin buildup with flushed, scale, skin, and plaques.

## Review

Psoriasis begins in one-third of the overall cases in childhood itself and is of long duration. It is a commonly occurring inflammation-related disorder of the layer of skin that is immune-mediated [5]. For the exacerbation and onset of the disease, there are numerous factors: mutations in the gene 14 of the recruitment of the caspase domain of the family and the genetic factor which has the HLA-Cw6; environmental factors like medications, lifestyle; and infectious diseases [6].

Some triggers like injury to the skin or medications like lithium, quinidine, antimalarial drugs, infections, and stress, cause most kinds of psoriasis. Allergies, weather, and diet too can be the other triggers for this condition. There are about seven main kinds of psoriasis: plaque-type psoriasis; Guttate psoriasis; inverse psoriasis; pustular psoriasis; erythrodermic psoriasis; nail psoriasis; and psoriatic arthritis.

While identifying this disease, we look for its symptoms, which can appear like rashes occurring in patches and look different as we see them in each individual. Some may appear as major eruptions all over the body or dandruff-like scaling. It could also be rashes with variation in the colours like shades of brown or pink or black skin or grey with purple or even with red along with silver scaling on the white skin, or cracked skin due to dryness that might bleed, or scaling small spots usually occurring in children, burning sensation, soreness, the appearance of episodic rashes that would aggravate for some weeks or months and then eventually subside.

Types of psoriasis

As we mentioned earlier, among the various kinds of psoriasis, plaque is one. It causes raised patches of skin covering scales, itchiness, and dryness, and it is the most common kind of psoriasis. Scalp, knees area, elbow, and lower back are the frequent occurring sites. Depending on the colour of skin, there is variation in the colour of patches. Particularly on brown or black skins, there might be temporary changes due to postinflammatory hyperpigmentation in the appearance of colour as an outcome of the healing of the altered part of the layer of skin.

In the other kind, we see nail psoriasis which causes abnormally grown nails with discolouration and pitting affecting the fingernails and toenails. The nails could loosen up and get separated from the nail bed in this, also called onycholysis, and if it gets severe, then the nails may even crumble.

Further, there is also Guttate and inverse psoriasis, wherein the prior one mainly affects children and among adults and is mainly triggered by any infection caused due to *Streptococcus, *which is a bacterial infection. It is identified by scaling spots all over the trunk or arms, and legs, which are small drop-shaped. Inverse psoriasis is another kind in which there is an occurrence of inflamed skin which appears in smooth patchwork and worsens with sweating and friction, and it commonly acts on the folding of the skin of the area of the groin or buttocks and also of breasts. This kind of psoriasis is usually triggered by fungal infections.

The very least occurring kind of psoriasis is the erythrodermic type psoriasis which may either be chronic, which is of longer duration, or acute, which is of short duration. It appears like a peeling form of a rash that can itch or burn covering the entire body surface.

A rare kind which can be defined as blisters with pus is pustular psoriasis. It can appear in the small area of the sole and palm or like widespread patches. The most clearly demarcated are the generalized pustular, palmoplantar, and acrodermatitis continua of Hallopeau among pustular psoriasis which is a heterogenous entity of different organ disease subtypes clinically. These are different from psoriasis vulgaris in phenotype and genetic ways but these subtypes may resemble to plaque psoriasis, establishing the rationale for the inclusion in the psoriasis band. As shown by the recent identification of mutation of three different kinds of genes, of the skin's innate immune systems, the genetic background is thought to be monogenic which is unlikely in psoriasis, the genes are IL36RN, CARD14 and AP1S3 [7]. Paradoxical psoriasis form of dermatitis is usually triggered by subtypes of generalized pustulars and its various kinds like acute pustulosis, acrodermatitis, pustular of palmoplantar, and different kinds of pustular of mostly a TNF-blocker. Table 1 gives the types of psoriasis [8].

**Table 1:** Types of Psoriasis and Their Characteristics

Types of Psoriasis	Its Characteristics
Plaque psoriasis	Itchy, dry, covered with scales
Nail psoriasis	Abnormal grown nails and discoloration
Guttate psoriasis	Scaling spots all over trunk, arm
Inverse psoriasis	Inflamed skin in smooth patchwork
Erythrodermic psoriasis	Peeling form of rash
Pustular psoriasis	Blisters with pus
Psoriatic arthritis	Arthritis associated

Pustular psoriasis and psoriatic arthritis

The pustular type of psoriasis may be present as the generalized type in the form of recurrent illness which is systemic, or as in palmoplantar type in the form of a locally centred disease mainly affecting the sole and palm, or in acrodermatitis in the nail beds or its digits. The consequences and severity should not be ignored or taken lightly, although these types of conditions are rare. With the capability of life-harming complications like a medical emergency of generalized pustular type of psoriasis when it appears like an acute episode like a flare. Debilitating conditions can be seen in the palmoplantar pustular type of psoriasis and in the acrodermatitis continua of Hallopeau. While in acrodermatitis there may be irreversible damage to the bone or nail, whereas in palmoplantar pustular psoriasis there is health-wise-related impaired life quality and morbidity psychiatrically [9].

**Figure 1:**
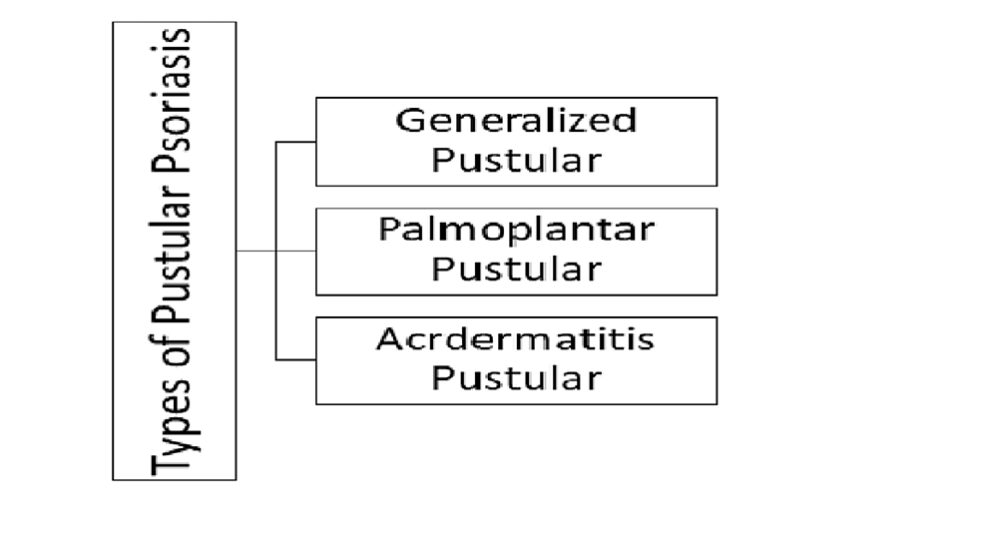
Types of Pustular Psoriasis Image Credit: Anushka Dhabale

Fever and malaise generally are accompanied by a systemic type of inflammatory, chronic disease, that is the generalized pustular type of psoriasis. Multiple pustules which are sterile occur all over the body surface along with diffused erythema and extremities swelled up, in generalized pustular psoriatic patients. There can be health-threatening situations as generalized pustular often reoccur in the lifetime. Clinicians and researchers are being provided with major advances in the approach towards the pathomechanism of generalized pustular understanding with the help of the underlying genetic molecular basis of different cases with recent discoveries. Figure 1 give the types of pustular psoriasis [10].

The discovered anomalies include an unusual gain of the function of mutations in gene encoding around keratinocyte signalling molecule CARD14 and a loss-of-function mutation in the interleukin 36 receptor antagonist gene. Neutrophils and interleukin 36 (IL-36) are now recognised as key players in the pathogenesis of generalized pustular, with IL-36 signalling serving as a connecting link between the responses of innate and adaptive immune systems. Inflammation is now thought to be brought on by an aberrant innate immune response that is primarily genetically determined and results in an inflammatory kind of keratinization. Currently, generalized pustular is regarded as a representative of this newly discovered class of skin disorders known as autoinflammatory keratinization disease [11].

Retinoids, or methotrexate, or cyclosporine, also corticosteroids, or TNF-alpha inhibitors, topical therapy, and phototherapy are among less well-established treatments. TNF-alpha inhibitors should be used in conjunction with methotrexate to prevent the development of antidrug antibodies [12].

Around 20% of patients referred to the early arthritis clinic have psoriatic arthritis, which is difficult to diagnose and treat. For the prevention of the function loss occurring long term and also to assure the best arthritis management and important comorbidities, early diagnosis is crucial. The differential diagnosis for a rheumatologist includes rheumatoid arthritis, also gout, including various inflammatorily arthritides. Once the condition has been identified, it is critical to thoroughly evaluate it, looking for signs of arthritis, or enthesitis, or dactylitis, or skin/nail disease, and also axial involvement [13]. 

Psoriatic arthritis is a chronic, autoimmune-mediated, inflammatory arthropathy that affects the joints and entheses, particularly those of the axial skeleton. It is associated with an increased risk of cardiovascular disease mortality [14].

Treatment of psoriasis 

Cytokine inhibitors, particularly those specific for tumour necrosis factor and, more recently, the interleukin 23-T-helper-17 cell pathway, have been very successful in the treatment of disease manifestations in a variety of tissues, even though targeting the interleukin 23-T-helper-17 cell pathway may be more effective in treating psoriasis than arthritis [14]. In Western adults, it is prevalent at 2-4%, and psoriatic arthritis develops in 20-30% of psoriasis sufferers [15]. This illness affects several organ systems, including skin, nails, entheses, peripheral and axial joints, and nails. Osteoporosis, or uveitis, or subclinical intestinal inflammation, and also cardiovascular disease are all associated with psoriatic arthritis as comorbidities. Its heterogeneity has made diagnosis challenging. Here, we review its classification criteria in an updated manner. CASPAR, which stands for Classified Criteria for Psoriatic Arthritis, type of screening instruments are used to help in quick diagnosis, recent discoveries on aetiology, and new therapy modalities, which also include biological drugs [15].

Historically, non-steroidal anti-inflammatory drugs and the same old medicines that treat rheumatic diseases were used to treat psoriatic arthritis patients. Although their ability to halt the radiological development of joint disease is not established. Contrarily, anti-tumour necrotic factor medications such as certolizumab, or etanercept, or infliximab, or adalimumab, and also golimumab are considered in this aspect. Apremilast, an orally taken phosphodiesterase 4 inhibitor, tofacitinib, a Janus kinase inhibitor, and numerous new biologics that target the IL-23 and IL-17 pathways, such as secukinumab, or brodalumab, or ixekizumab, and also ustekinumab, are among the latest psoriatic arthritis medications [16].

Evidence suggests nutrition performs a significant aspect in the aetiology of psoriasis which is growing, among other psoriasis risk factors. In particular, diet, nutrition, and body weight may worsen or possibly start the disease's clinical signs [17]. There are a number of reasons that could account for the elevated frequency of cardiovascular events in the psoriasis population. The high prevalence of traditional cardiovascular risk factors and metabolic disorders are the main contributors to the significant cardiovascular burden in psoriasis patients. Similarly, the coexistence of systemic inflammation and metabolic disorders may raise the risk of cardiovascular disease in these people [18].

Psoriasis vulgaris is the most well-known and manageable human disease that is mediated by T lymphocytes and dendritic cells. Inflammatory myeloid dendritic cells release IL-23 and IL-12 to encourage IL-17-producing T cells, Th1 cells, and Th22 cells to produce significant amounts of the psoriatic cytokines IL-17, IFN (interferon), TNF, and IL-22 [19]. Patients with this genotype have been observed to have distinct clinical characteristics and acquire the disease at an earlier age, with a concordance of about 60% in monozygotic twins. HLA-Cw*0602 is a substantial risk factor for the beginning of the illness, and homozygous people are also at risk, according to recent linkage and higher resolution association studies.

Compared to heterozygotes, they have a disease risk that is around 2.5 times higher for this gene. According to published evidence, (cells of differentiation) CD8+ T cells may be a key effector in psoriasis. A notable characteristic of persistent psoriasis lesions is epidermal infiltration of oligoclonal CD8+ T cells, which are reacting to particular antigens, and likely also of CD4+ T cells in the dermis [20].

Local treatments, or phototherapies, and also systemic treatments like standard systemic therapy and biotherapy, are all currently available and, in the major part of the cases, are sufficient to control this skin condition. So as to improve these children's lifetime, subsequent management should concentrate on preserving therapeutic efficacy and preventing recurrence by minimising any of it [21]. 

Hydration of skin like frequent use of moisturisers and emollients, careful, and gentle skin cleaning, detection and avoidance of triggers related to the phenomenon of Koebner like excoriation, maceration, and foci which are infectious are all important parts of treating psoriasis (*Streptococcus pyogenes*). Patients with psoriasis have shown that moisturisers considerably reduce their skin problems and enhance their quality of life. Due to the prevalence of dry skin, which increases the irritation of sick skin, they are an effective first-line treatment [22].

Newer topical treatments like calcipotriol and immunosuppressive medications like cyclosporin A and FK506 are significantly changing how psoriasis is treated [23]. Up until recently, corticosteroids, tars, anthralins, and keratolytics were the cornerstones of topical therapy. However, recently, topical retinoids, a novel anthralin preparation, and vitamin D analogues have increased doctors' treatment toolkits [24].

The topical management of psoriasis requires the use of emollients, moisturisers, and keratolytic medications. They serve as adjuvants to conventional therapies and aid in lowering the scale load of particular patients. Emollients and moisturisers primarily function to support proper hyperproliferation, or differentiation, and apoptosis; additionally, they have anti-inflammatory actions, for instance through physiologic lipids [25].

Upper respiratory tract infection is the most common reason for asthma in children. Treatment is determined based on the disease's severity and whether or not it has affected any joints. Corticosteroids and calcipotriene are examples of topical treatments. Systemic retinoids, ultraviolet radiation, and cyclosporine all reduce cutaneous psoriatic lesions. Both the cutaneous and joint symptoms of psoriasis respond well to methotrexate sodium and etanercept [26]. People with more severe, persistent, or extensive psoriasis can benefit from systemic medications, phototherapy, and other treatments. Although these treatments are more efficient than topical ones, they are also linked to serious cutaneous and systemic side effects [27].

UVB, that is ultraviolet B phototherapy, is a successful treatment for the widespread disease that allows for both quick management and long-term maintenance [28]. While cyclosporine is helpful, especially when used briefly in acute exacerbation situations, it should be substituted by other treatments for long-term maintenance [28]. Lower concentrations and shorter durations of topical corticosteroids should be prescribed for treating children. Patients who are pregnant or nursing can benefit from topical corticosteroids in a safe and efficient manner. They are available in many different formulations, including shampoos, ointments, creams, lotions, gels, foams, and oils [29]. Although topical steroids are often used, there are only a few disorders that have been proved to benefit from their usage, such as psoriasis, vitiligo, eczema, atopic dermatitis, phimosis, acute radiation dermatitis, and lichen sclerosus [30]. 

Diet and psoriasis

The likelihood of psoriasis symptoms improving appears to be higher for foods and substances with systemic anti-inflammatory properties [31]. When combined with topical or systemic therapy, a low-calorie diet (LCD) improves the Dermatology Life Quality Index and Psoriasis Area and Severity Index. However, LCD was not successful in maintaining disease remission when patients stopped concurrent cyclosporine or methotrexate therapy [32]. Psoriasis patients usually have an imbalanced diet, with a higher consumption of fat and a lower intake of fish or dietary fibre, as compared to controls. Such dietary habits may have an impact on the frequency and intensity of psoriasis. Nutrition has an impact on the start, progression, and comorbidities of psoriasis [33]. Body mass index and psoriasis severity have been linked in various studies, and obesity has been linked to a pro-inflammatory condition [34].

COVID-19 and psoriasis

When it comes to the safety and effectiveness in patients having covid vaccine with immune-mediated inflammatory diseases (IMIDs), there is little reason to believe that these patients face any higher risk of negative side effects than healthy controls [35]. Because of the elevated risk of infection, especially in high-risk areas, conventional immunosuppressive medications like methotrexate and cyclosporine, as well as anti-TNF drugs, should not be recommended. The side effect of hypertension, which has been linked to a higher likelihood of developing severe COVID-19 (coronavirus disease), may make using cyclosporine riskier. Given the lack of conclusive evidence to date that biologics increase the risk of COVID-19, these drugs should only be stopped when a patient displays COVID-19 symptoms [36]. Due to the COVID-19 pandemic, clinicians treating IMIDs, such as psoriasis, have encountered significant challenges. Patients with severe psoriasis are more likely to have obesity, hypertension, diabetes, and male sex as risk factors for severe COVID-19. The risk of severe infection is also known to increase with the use of several systemic psoriasis treatments. Therefore, it makes sense that in the early stages of the pandemic individuals receiving typical targeted systemic medication were believed to have a greater chance of getting a severe COVID-19 infection. In addition to risk-reducing behaviours like social distance suggested by the World Health Organization, people who were deemed to be more sensitive, such as those using immunosuppressants, were encouraged to adopt greater measures of social isolation [37]. The COVID-19 pandemic negatively affects the treatment of psoriasis and the provision of healthcare [38]. Patients with psoriasis who have had biological treatment or another sort of systemic therapy may develop a mild case of SARS-CoV-2 (severe acute respiratory syndrome coronavirus 2) infection, while they may also briefly experience an aggravation of skin lesions [39].

## Conclusions

Skin disease known as papulosquamous, formerly thought to be primarily an epidermal keratinocyte issue, psoriasis is now known to be one of the most common immune-mediated disorders. It comes in a variety of forms, including plaque, pustular, and more. To better understand current trends in psoriasis in general, we reviewed recent advancements in psoriasis epidemiology, aetiology, and genetics. In this review, we have also discussed the relation between psoriasis and diet, COVID-19, and its treatment. We have described the kinds of psoriasis with their characteristics and seen that an appropriate diet can help to maintain the symptoms of psoriasis. And COVID-19 has a negative impact on the treatment of psoriasis.
